# Low pH condition impairs BP‐IgG binding to the basement membrane zone

**DOI:** 10.1111/1346-8138.17175

**Published:** 2024-03-14

**Authors:** Dongjun Im, Keisuke Ueda, Hirofumi Niwa, Kayoko Tanaka, Hiroaki Iwata

**Affiliations:** ^1^ Department of Dermatology Gifu University Graduate School of Medicine Gifu Japan

**Keywords:** autoantibody, bullous pemphigoid, direct immunofluorescence, inflammation, pH

## Abstract

Bullous pemphigoid (BP), an autoimmune subepidermal blistering disease, shows tense blisters associated with urticarial erythema. Tissue‐bound Immunoglobulin G (IgG) at the basement membrane zone (BMZ) detected by direct immunofluorescence (DIF) is strong evidence for a diagnosis of BP. The sensitivity of DIF is higher in complement component 3 (C3) than in IgG, but the reason for this different sensitivity is not fully understood. In this study, we performed several ex vivo studies to investigate the possible mechanism of IgG negativity and C3 positivity at the BMZ by DIF in some BP cases. First, sera from BP patients showing IgG negativity by DIF were found to clearly react to the BMZ in their own DIF skin samples. Next, indirect immunofluorescence (IIF) was performed using sera diluted with different pH phosphate‐buffered saline (PBS), pH 7.4, 6.0, and 3.0. Patients' sera diluted with pH 7.4 PBS showed linear staining at the BMZ, but sera diluted with pH 6.0 PBS and pH 3.0 PBS showed lower fluorescence intensities. Finally, sections of skin from BP patients were pre‐incubated with different pH PBS (pH 3.0, 6.0, and 7.4), followed by staining with anti‐human IgG and C3. The fluorescence intensities were notably lower for IgG and C3 that had been pre‐incubated with pH 3.0 PBS and pH 6.0 PBS than for IgG and C3 that had been pre‐incubated with pH 7.4 PBS. These results suggest that a low pH condition hinders the binding of autoantibodies to the BMZ, that is, the drop in tissue pH induced by inflammation inhibits autoantibodies from depositing at the BMZ. Furthermore, the drop in tissue pH causes tissue‐bound autoantibodies to detach from the BMZ. Complement fragments are activated not only on IgG but also on the cell surface of cells close to IgG during complement activation. IgG may detach from the BMZ under low pH condition induced by inflammation, but some complement fragments remain at the BMZ. These phenomena may help to explain why C3 is more sensitive than IgG when DIF is used to diagnose BP.

## 1 INTRODUCTION

Bullous pemphigoid (BP), an autoimmune subepidermal blistering disease, clinically presents as tense blisters associated with urticarial erythema.^1^ Histologically, subepidermal separation at the basement zone (BMZ) is associated with inflammatory infiltrates, mainly eosinophils. Patients have autoantibodies against two major hemidesmosomal molecules^1^: type XVII collagen (BP180, also known as BPAG2)^2^ and BP230 (also known as BPAG1).^3^ Direct immunofluorescence (DIF), which shows tissue‐bound antibodies, is strong evidence for the diagnosis of BP. In typical BP cases, DIF shows linear deposits of IgG and complement component 3 (C3) at the BMZ. It is recommended that the biopsy for DIF should be taken from perilesional or normal‐appearing skin, and not from a blister lesion^4^, ^5^, ^6^, ^7^ because autoantigens can degrade around blister lesions. In mucous membrane pemphigoid, DIF on biopsies from non‐lesional mucosa is similarly useful.^8^, ^9^ In addition, C3 is known to have higher sensitivity than IgG in DIF.^10^, ^11^ The reason for the different sensitivity between IgG and C3 is not fully understood.

Antibody–antigen interactions are mediated by several mechanisms, such as electrostatic interactions, hydrogen bonding, and hydrophobic interactions. It is well known that extreme pH conditions, such as pH below 6.5 or above 8.5, tend to interfere with antibody–antigen interactions, especially electrostatic interactions and hydrogen bonding.^12^ In addition, pH control tends to be essential for maintaining tissue homeostasis in the human body. Tissue pH fluctuates under several conditions, such as malignant neoplasms, inflammation, and ischemia.^13^, ^14^ For example, in local tissues, ischemia followed by inflammation causes the pH to fall to 6.5–6.0.^15^ Furthermore, a low pH condition from inflammation results in chronic inflammation. In arthritis, the synovial fluid has a lower pH (6.8–7.1) than that in the normal condition (pH 7.4–7.8).^16^ Psoriasis is a chronic inflammatory skin disease, and BP is also frequently associated with urticarial erythema, which suggests local inflammation. No previous reports have investigated local tissue pH in psoriasis or BP, but we expected the pH of affected skin to be lower than normal.

Based on the above evidence, we hypothesized that a low pH condition hinders autoantibodies from binding to autoantigens. That is, autoantibodies in BP patients bind to autoantigens and, after inflammation induced by complement activation, the autoantibodies detach from the autoantigens, or the autoantibodies fail to bind to autoantigens in inflammatory tissue such as that of psoriasis. In this study, we investigated the impact of pH on IgG binding at the BMZ in BP patients.

## 2 MATERIALS AND METHODS

### 2.1 Patients

The BP patients were diagnosed according to the following criteria: (i) clinical blistering or erosions on the skin, (ii) IgG and/or C3 deposits at the BMZ shown by DIF, and (iii) autoantibodies detected by BP180‐NC16A chemiluminescence enzyme immunoassay (CLEIA) (MBL, Nagoya, Japan). Four BP patients from each of two different groups were enrolled: DIF‐positive for only C3 (Table 1, nos 1–4), or DIF‐positive for both IgG and C3 (Table 3, nos 5–8). Clinical phenotype and histological inflammatory severity are shown in Tables 1 and 3. Skin for DIF was taken from the perilesional area. This study was approved by the Gifu University Certified Review Board and was performed in accordance with the Declaration of Helsinki (#2022–155).

**TABLE 1:** Summary of the results for patients who showed DIF IgG−/C3+.

No.	Clinical type	Histological inflammation	DIF IgG	DIF C3	BP180‐NC16A CLEIA	IIF DIF skin	mAb^a^ DIF skin
1	Non‐iflammatory	Severe	−	1+	87.5	1+	2+
2	Inflammatory	Moderate	−	2+	68.4	1+	1+
3	Inflammatory	Moderate	−	1+	9370	2+	1+
4	Inflammatory	Moderate	−	2+	95.5	2+	2+

*Note*: The serum anti‐BP180‐NC16A IgG titer level was measured by CLEIA (normal <9.0 U/mL).

Abbreviations: CLEIA, chemiluminescent enzyme immunoassay; DIF, direct immunofluorescence; IgG, immunoglobulin G; IIF, indirect immunofluorescence; mAb, monoclonal antibody.

^a^ hBP180‐NC16A mAb.

#### 2.1.1 Preparation of PBS with three different pHs

In this study, we used phosphate‐buffered saline (PBS) with three different pHs (pH 7.4, 6.0, and 3.0) without calcium. To prepare these buffers, 10× PBS buffer (26.8 mM KCl, 81.0 mM Na_2_HPO_4_, 14.7 mM KHPO_4_, 1.54 M NaCl) was diluted with 900 mL of distilled water and the pH was adjusted to 3.0, 6.0, or 7.4 using 1–6 N HCl. The final volume was brought up to 1000 mL.

#### 2.1.2 Recombinant mAbs against BP180‐NC16A

Recombinant human IgG1 monoclonal antibodies (mAbs) targeting BP180‐NC16A (hBP180‐NC16A mAb) were produced by HEK293 cells transfected with heavy‐chain and light‐chain vectors. The sequence of variable regions [variable region of light chain (VL) and variable region of heavy chain (VH)] targeting BP180 NC16A amino acids 514–520 from human BP was previously reported.^17^ The synthesized genes of the heavy chain of human IgG1, containing VH, and of the light chain of human IgG1, containing VL, were cloned into the BamH1 and EcoRI restriction enzyme sites of the pcDNA3.1(+) vector. The pair vectors of the heavy chain and the light chain were co‐transfected into HEK293T cells using the lipofectamine3000 transfection reagent (Thermo Fisher Scientific, Waltham, MA, USA) according to the manufacturer's instructions. The transfected cells were maintained in Dulbecco's modified Eagle medium (Life Technologies, Tokyo, Japan) without fetal bovine serum. IgG was purified from the culture supernatant using the Protein G Column (GE Healthcare, Chicago, IL, USA) according to manufacturer's instructions.

#### 2.1.3 Modified DIF and IIF tests

Skin from normal subjects and from BP patients was frozen in liquid nitrogen, and 5‐μm‐thick sections were prepared by cryostat (Leicabiosystems, Tokyo, Japan). For the DIF test on skin from patients (DIF‐positive in both IgG and C3), sections were incubated with PBSs of different pHs (pH 7.4, 6.0, and 3.0) for 30 min at 37°C. After being washed with PBS (pH 7.4), the sections were incubated with fluorescein isothiocyanate (FITC)‐conjugated anti‐human IgG (1:100; DakoCytomation, Glostrup, Denmark) and anti‐human C3 (1:100; DakoCytomation) for 45 min at 37°C.

For the indirect immunofluorescence (IIF) test, skin from normal humans and skin from patients (DIF‐positive in only C3) was stained with patients' sera (1:20 dilution in PBS, pH 7.4, 6.0, and 3.0) and with hBP180‐NC16A mAbs as the primary antibodies for 45 min at 37°C, followed by incubation with FITC‐conjugated anti‐human IgG (1:100; DakoCytomation) as the secondary antibodies for 30 min at 37°C. In the IIF using DIF samples, serum was used from the same patient as for the DIF skin samples.

#### 2.1.4 Image analysis

Florescent images were taken with a Keyence fluorescence microscope (Keyence, Osaka, Japan). For semiquantitive evaluation, the fuorescence intensity at the BMZ was defined as 2+ for clear linear deposits, 1+ for linear deposits, ±for visible linear deposits, and −for no lienar deposits.

## 3 RESULTS

### 3.1 BP180 is clearly detected in BP patient skin that tested IgG‐negative by DIF

**FIGURE 1:**
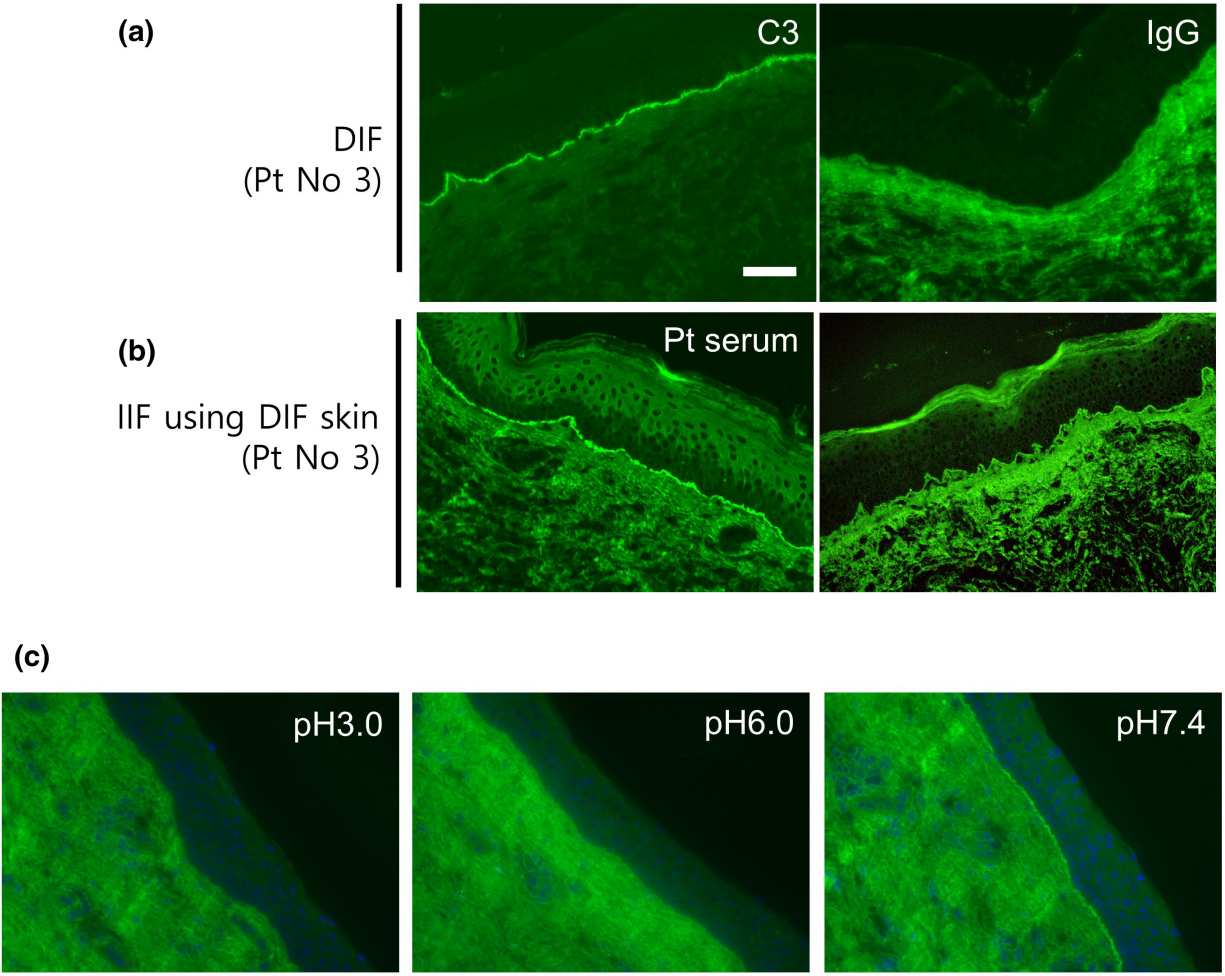
Indirect immunofluorescence (IIF) using serum of patient with IgG negativity and C3 positivity. (a) Representative direct immunofluorescence (DIF) images of C3 (left) and IgG (right). (b) Incubation with patient serum (left) or hBP180‐NC16A monoclonal antibodies (right) using DIF skin. (c) IIF studies were performed using sera diluted with phosphate‐buffered saline of pH 3.0, 6.0, or 7.4. Scale bar: 100 μm. Representative images for number 3 in Table 2.

To investigate whether the BP180 autoantigen was present in the patients' DIF skin samples, we recruited four BP patients (nos 1–4) who had tested IgG‐negative by DIF (Figure 1a). The patients' sera clearly reacted to the BMZ in IIF using normal human skin. In addition, anti‐BP180‐NC16A IgG was elevated (68.4–9370 U/mL; Table 1). These results indicate that the patients had autoantibodies against the BMZ, including anti‐BP180‐NC16A IgG.

Next, IIF was performed using DIF skin samples as the substrate to investigate whether autoantigens at the BMZ were present in the patients' DIF skins. All four sera from BP patients that tested IgG‐negative by DIF also reacted to the BMZ of their own DIF skin (Table 1 and Figure 1b). In addition, the hBP180‐NC16A mAb reacted to the BMZ in all DIF skins (Figure 1b). These results suggest that autoantigens are present at the BMZ even in the patients that tested IgG‐negative by DIF.

### 3.2 Low pH condition inhibits BP‐IgG biding to the BMZ by IIF

Next, we investigated the impact of pH on the autoantibody binding efficacy in IIF. The semiquantitative evaluation of linear staining at the BMZ is summarized in Table 2. Patients' sera diluted with pH 7.4 PBS showed clear staining at the BMZ (Figure 1c, right). In contrast, sera diluted with pH 6.0 PBS showed lower fluorescence intensity (Table 2: 1 for 1+, 2 for ± and 1 for −; Figure 1c: middle). Sera diluted with pH 3.0 PBS showed almost no reactivity (Table 2: 1 for ± and 3 for −; Figure 1c: left). Anti‐BP180‐NC16A mAbs showed similar reactivities: positive for pH 7.4 PBS, weakly positive for pH 6.0 PBS, and negative for pH 3.0 PBS (Table 2). Representative images are shown in Figure 1c.

**TABLE 2:** Summary of the results of IIF incubating sera or hBP180‐NC16A mAbs diluted with PBS at different pHs.

No.	pH 3.0	pH 6.0	pH 7.4
1	−	±	1+
2	−	1+	1+
3	−	±	1+
4	±	±	1+
hBP180‐NC16A mAb	−	±	1+

Abbreviations: IIF, indirect immunofluorescence; mAb, monoclonal antibody; PBS, phosphate‐buffered saline.

### 3.3 At low pH, BP‐IgG detaches from the patient's skin

**FIGURE 2:**
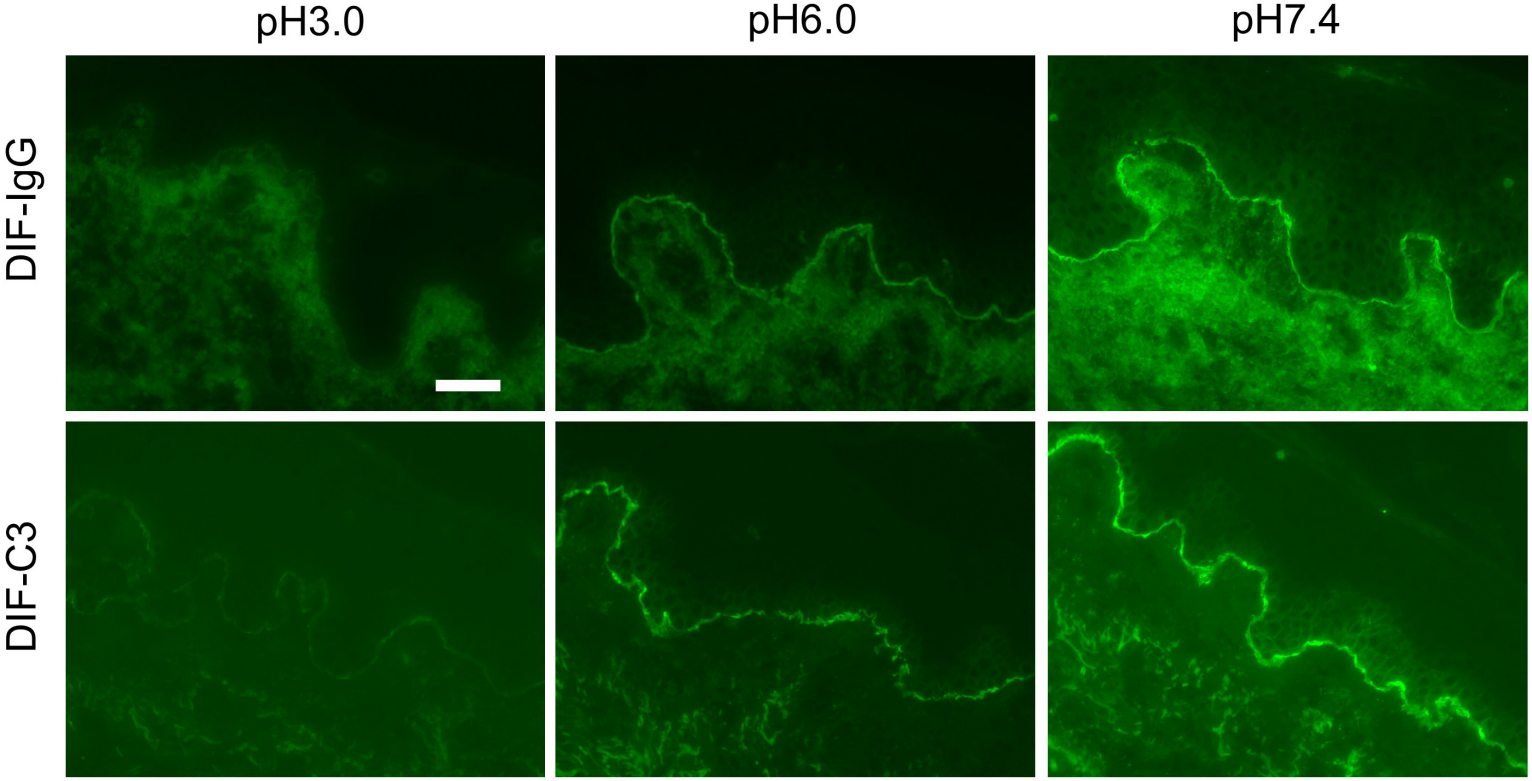
Direct immunofluorescence (DIF) samples pre‐incubated with phosphate‐buffered saline (PBS) of different pHs. DIF samples were pre‐incubated with PBS of pH 3.0, 6.0 or 7.4, followed by staining with FITC‐conjugated anti‐human IgG or anti‐human C3. Scale bar: 100 μm. Representative images for Pt. Number 7 in Table 3.

Finally, we investigated whether IgG detaches from the BMZ of the patient's skin under acidotic conditions. DIF tests were performed with modifications. Skin sections from patients (nos 5–8) that showed positivity for IgG and C3 at the BMZ by DIF were pre‐incubated with PBS of different pHs (pH 3.0, 6.0, and 7.4), followed by staining with anti‐human IgG and C3. The fluorescence intensities of IgG and C3 pre‐incubated with pH 3.0 PBS and pH 6.0 PBS were obviously lower than those pre‐incubated with pH 7.4 PBS (Table 3). Representative images are shown in Figure 2. Under the pH 3.0 condition, IgG stained almost not at all, but C3 stained faintly.

**TABLE 3:** Summary of the results of DIF for pre‐incubation with PBS at different pHs.

No.	Clinical type	Histological inflammation	DIF	pH 3.0	pH 6.0	pH 7.4
5	Inflammatory	Severe	IgG	−	1+	1+
			C3	±	1+	2+
6	Inflammatory	Moderate	IgG	1+	1+	2+
			C3	±	1+	2+
7	Inflammatory	Mild	IgG	−	1+	1+
			C3	±	1+	2+
8	Inflammatory	Mild	IgG	−	1+	2+
			C3	±	1+	2+

Abbreviations: C3, complement component 3; DIF, direct immunofluorescence; IgG, Immunoglobulin G; PBS, phosphate‐buffered saline.

## 4 DISCUSSION

This study demonstrates that a low pH condition hinders autoantibodies from binding to the BMZ in an ex vivo assay. These results suggest two possible behaviors of autoantibodies in BP blistering. One is the possible disruption of autoantibody binding to the BMZ caused by acidotic conditions, such as in skin inflammation. The other is the detachment of tissue‐bound autoantibodies at the BMZ that may occur under acidotic conditions. The detachment of autoantibodies from urticarial lesions of BP skin leads to a higher sensitivity of C3 than of IgG in DIF tests on BP patients.

Negativity for IgG in DIF sometimes occurs in BP. The sensitivity of DIF differs between IgG and C3, with previous reports showing C3 to have a higher sensitivity.^10^, ^11^ The reason for the different sensitivities is unclear, but the degeneration of autoantigens is generally considered to be involved. To make this discrepancy clear, we first performed antigen mapping in patients' DIF skin samples. Interestingly, sera from BP patients who demonstrated IgG negativity and C3 positivity by DIF visibly reacted to the BMZ of their own skin sections. This result clearly indicates that autoantigens of the BMZ exist even in IgG‐negative DIF patients' skin. In addition, anti‐human BP180‐NC16A mAbs also were able to show linear staining, suggesting that BP180 autoantigens are not degraded. In other words, patients have autoantibodies against the BMZ in their sera, and these autoantibodies can be detected by IIF as binding to the BMZ. Nevertheless, why is it that patients' autoantibodies sometimes cannot be detected by DIF?

We hypothesized that inflammation may interfere with IgG binding to the BMZ. When considering how inflammation prevents such binding, we focused on the drop in pH in inflammatory tissues. To confirm this speculation, IIF was performed using sera diluted with buffers of different pHs (pH 3.0, 6.0, and 7.4). IgG bound less readily to the BMZ for the low pH conditions of pH 6.0 and pH 3.0 than for the physiological condition of pH 7.4. These results indicate that localized low pH due to inflammation can hinder autoantibodies from binding to the autoantigens at the BMZ. However, the discrepancy in the sensitivity of IgG and C3 by DIF is still unclear. We speculated that tissue‐bound IgG may detach by inflammation.

To confirm this speculation, we performed DIF tests after incubation under different pH conditions. As we expected, low pH conditions decreased the fluorescence intensity of both IgG and C3 at the BMZ. Pre‐incubation with pH 3.0 PBS resulted in almost no IgG deposits, but in faint C3 deposits at the BMZ. In the classical complement activation cascade, C1q starts the activation cascade by initially binding to the CH2 domain of IgG or IgM of immune complexes.^18^, ^19^ Three complement cascades lead to the activation of C3 convertase, and then this enzyme cleaves C3 into anaphylactic peptide C3a and opsonin C3b. The C3b generates C5 convertase, and then the C5 cleaves into C5a and C5b. The C5b finally forms C5b‐9 complexes, which can cause cell lysis. Anaphylactic C3a and C5a can induce inflammation via the recruitment of neutrophils or macrophages, and this complement activation play a crucial role in the blistering mechanism of BP.^20^ In this cascade, complement fragments are activated not only on IgG but also on the cell surface close to IgG. In other words, some complement fragments exist apart from the IgG immune complex. Although IgG detaches from the BMZ by inflammation, some complement remains at the BMZ. According to our results, DIF treated with the low‐pH condition showed reduced fluorescence intensity for both IgG and C3, with almost no staining of IgG and only faint staining of C3 at pH 3.0. These mechanisms could be one reason that C3 has higher sensitivity than IgG by DIF in some BP cases.

This study had some limitations. We did not show tissue pH changes in inflammatory skin, including in urticarial erythema of BP. In addition, most of the patients in this study had the inflammatory phenotype. In the future, we would like to investigate the pH changes in vivo, and to compare IgG deposits between inflammatory lesions and non‐inflammatory lesions.

**FIGURE 3:**
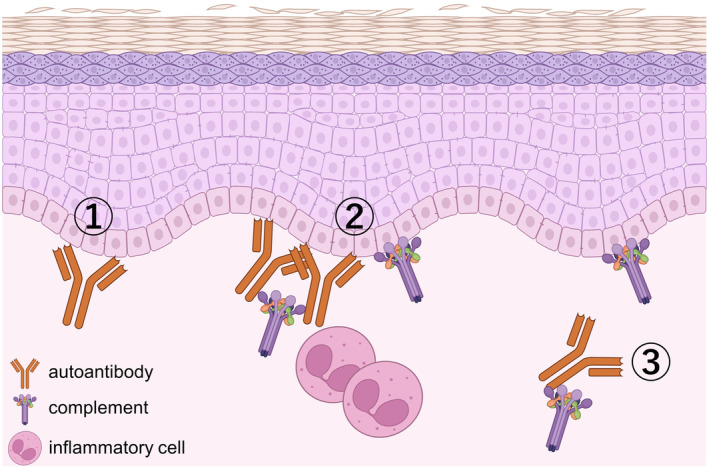
First, autoantibodies bind to the basement membrane zone (BMZ), and then complement cascades are activated on IgG and on the surface of basal cells. Inflammatory cells are recruited and activated, and the tissue pH decreases. Finally, tissue‐bound autoantibodies detach from the BMZ, but some of the compliment remains on the basal cell surface. Created with BioRender.com.

In summary, autoantibodies first bind to the BMZ (Figure 3, left) and activate complement cascades (middle). The activated complements induce inflammation by recruiting inflammatory cells (Figure 3, middle). The inflammation leads to a drop in tissue pH, and the low pH condition hinders BP‐IgG from binding to the BMZ. Furthermore, the drop of tissue pH may cause tissue‐bound autoantibodies to detach from the BMZ (Figure 3, right). This may be associated with the higher sensitivity of C3 than IgG by DIF during the diagnosis of BP.

## CONFLICT OF INTEREST STATEMENT

None.
